# The Therpeutical Applications of Peroxide of Hydrogen

**Published:** 1895-05

**Authors:** 


THE THERAPEUTICAL APPLICATIONS OF PEROXIDE OF HYDROGEN, GLYCOZONE, AND HYDROZONE. By Charles Marchand, Chemist. Graduate of the "École Centrale des

Arts et Manufactures de Paris" (France). Ninth Edition. New York: 28 Prince Street, 1895.

This book has been reviewed in these pages before. It contains a full account of Peroxide of Hydrogen, and should be in the hands of every physician. It contains 200 pages and is illustrated by wood cuts. It will be sent free of charge to any physician who writes for it giving his full address and mentioning this journal.

